# Population Estimates and Hypertension and Diabetes Prevalence: Cross-Sectional Quantitative Study Comparing Electronic Health Record–Derived Counts, Census, and Centers for Disease Control and Prevention Population Level Analysis and Community Estimates

**DOI:** 10.2196/86337

**Published:** 2026-05-07

**Authors:** Tyler NA Winkelman, Kelly Bergmann, Peter Bodurtha, Alanna M Chamberlain, R Adams Dudley, David Haynes, Steven G Johnson, Thomas E Kottke, Karen L Margolis, Gabriela Vazquez Benitez, Devon Nerstad, Patrick Olson, James M Peacock, Nayanjot Kaur Rai, Stephen C Waring, Bjorn Westgard, Paul Englund Drawz

**Affiliations:** 1 Hennepin Healthcare Research Institute Minneapolis, MN United States; 2 Children's Minnesota Minneapolis, MN United States; 3 Mayo Clinic Rochester, MN United States; 4 University of Minnesota Minneapolis, MN United States; 5 HealthPartners St Paul, MN United States; 6 Healthy Quin Counties Thief River Falls, MN United States; 7 Minnesota Department of Health St Paul, MN United States; 8 Essentia Health Duluth, MN United States

**Keywords:** prevalence, population, hypertension, diabetes, census

## Abstract

**Background:**

Accurate small-area estimates of vaccination rates and disease burden can inform public health interventions.

**Objective:**

This study aimed to compare population denominators derived from census data and electronic health record (EHR) data from a statewide collaboration in Minnesota and examine concordance between Centers for Disease Control and Prevention and EHR-based estimates of diabetes and hypertension prevalence at the census tract level.

**Methods:**

A retrospective study was conducted using EHR data from 2018 to 2022 from the Minnesota EHR Consortium (MNEHRC), population estimates from the 2020 census, and disease prevalence estimates among adults from the Centers for Disease Control and Prevention Population Level Analysis and Community Estimates (PLACES) project. Patients were included if they had a Minnesota address and a clinic visit in the last 3 years. Patients with hypertension and diabetes were identified based on the presence of at least 1 diagnosis code in the Observational Medical Outcomes Partnership condition occurrence table in the last 5 years or an elevated outpatient blood pressure (systolic blood pressure ≥140 mm Hg or diastolic blood pressure ≥90 mm Hg) on 2 or more days in the last 3 years for hypertension or at least 1 hemoglobin A_1c_ value of ≥6.5 in the last 3 years for diabetes.

**Results:**

The 2020 census estimate for the population of Minnesota was 5,707,254. A total of 5,271,191 (92.4% of the census estimate) unique individuals visited 1 of the 11 MNEHRC health care systems in 3 years (2018-2020). The ratio of MNEHRC patients to the Minnesota statewide 2020 census estimate was higher for female individuals (0.97) than for male individuals (0.88) and higher for older age groups (individuals aged 65 years and older: 1.05) than for younger age groups (individuals aged 0-17 years: 0.83). The MNEHRC patient-to-census ratio also differed by race—the ratio was the highest for Black Minnesotans (1.17) and the lowest for American Indian and Alaska Native Minnesotans (0.68). According to MNEHRC data, the percentage of adults in Minnesota with diabetes in 2022 was 9.5% (415,914/4,376,805), and the percentage of adults in Minnesota with hypertension in 2021 was 32.2% (1,365,413/4,234,000). Estimates from PLACES for diabetes were 9.9% (435,481/4,389,028) and for hypertension were 29.9% (1,311,459/4,389,028). The percentage of census tracts where the MNEHRC estimate was within 10% of the PLACES estimate was 40.3% (605/1500) for diabetes and 42.3% (635/1500) for hypertension; 77.9% (1168/1500) of census tracts for diabetes and 79.7% (1195/1500) for hypertension were within 25% agreement.

**Conclusions:**

Our analysis suggests that there are both similarities and important differences between small-area estimates derived from EHR and survey data. Such differences suggest that further research is needed to determine the optimal collection method for local estimates of health conditions.

## Introduction

Accurate population-level denominators are essential for providing reliable estimates of disease burden and evaluating public health interventions [1]. Many governmental and academic entities rely on census-derived population estimates to determine the rate of procedures and prevalence of chronic diseases [2,3]. These estimates of condition prevalence can guide intervention efforts to improve health at the neighborhood, state, and national levels [4-6]. However, inaccuracies in census data and discrepancies between census data and health data measures—particularly among underrepresented populations—have at times led to anomalies in reporting [7]. For example, during the COVID-19 pandemic, vaccination rates exceeding 100% were reported in some groups in both California and Massachusetts [8,9]. In Minnesota, we developed a statewide distributed data network that captures electronic health record (EHR) data for more than 90% of the population, offering an alternative source for deriving population-level denominators for public health data [10-13].

In addition to syndromic surveillance, population estimates play a critical role in allocating public health resources and designing interventions to address inequities [14]. The Centers for Disease Control and Prevention (CDC), through the Behavioral Risk Factor Surveillance System (BRFSS) and its Population Level Analysis and Community Estimates (PLACES) project, reports estimates of chronic disease prevalence for adults aged 18 years and older at the census tract level using small-area modeling that incorporates survey and census data [15]. While these modeled estimates guide federal and state funding to address health disparities, their accuracy may be limited by sparse sampling at the neighborhood level. On average, BRFSS includes only approximately 5 individuals per census tract per year [16]. By contrast, our EHR-based system enables direct observation of diagnosed chronic disease prevalence in nearly every census tract across Minnesota, offering an opportunity to compare EHR-based and model-based small-area estimates [10].

In this study, we compared population denominators derived from census data and Minnesota-based EHRs and examined concordance between CDC and EHR-based estimates of hypertension and diabetes prevalence at the census tract level in the adult population. We assessed concordance between CDC-modeled and EHR-derived prevalence estimates across Minnesota and explored the area-level sociodemographic factors associated with prevalence estimates from EHR data and CDC PLACES. These findings have implications for improving the accuracy of population health surveillance and ensuring equitable distribution of public health resources.

## Methods

### Minnesota EHR Consortium

The Minnesota EHR Consortium (MNEHRC) is a collaboration among individuals from the 11 largest health care systems in Minnesota, the Minnesota Department of Health, local public health departments, the University of Minnesota, and Minnesota Community Measurement. Each of the 11 health care systems has mapped its EHR data to the Observational Medical Outcomes Partnership (OMOP) common data model [17]. Patient addresses have been geocoded to census tracts to facilitate area-level analyses. Individuals who have sought care at multiple health systems are deduplicated using a secure, privacy-preserving record linkage process with hashed identifiers from each health care system [18,19]. An algorithm based on frequency of care in the last 3 years assigns individuals to 1 health care system for reporting purposes.

Data are from the Health Trends Across Communities (HTAC) in Minnesota project, which includes EHR-derived prevalence estimates for several chronic conditions at the census tract level. Patients are eligible for inclusion for a given year if they are alive and aged 18 years or older on December 31 of that year and have had an encounter in their assigned health care system in the past 3 years. Therefore, the EHR data represent a health care–seeking population. Patients with hypertension were identified based on the presence of at least 1 hypertension diagnosis code in the OMOP condition occurrence table in the last 5 years or an elevated outpatient blood pressure (systolic blood pressure ≥140 mm Hg or diastolic blood pressure ≥90 mm Hg) on 2 or more days in the last 3 years. Patients with diabetes were identified based on the presence of at least 1 diabetes diagnosis code in the last 5 years or at least 1 hemoglobin A_1c_ value of ≥6.5 in the last 3 years. To compare with census data, we used 2020 counts for population estimates (individuals alive on December 31, 2020, with an encounter in 2018-2020). To evaluate concordance with the most recent data available from CDC, 2022 data were used for counts of diabetes, and 2021 data were used for counts of hypertension.

### Census and CDC PLACES Estimates

Census data at the county level were obtained from the 2020 Census Demographic and Housing Characteristics Person and Housing tables [20]. Race and ethnicity data were grouped to be consistent with mutually exclusive categories in the EHR data: Hispanic and then non-Hispanic American Indian; Asian or Native Hawaiian or Other Pacific Islander; Black; White; and other (more than 1 race or other).

Census tract estimates of diabetes and hypertension prevalence among adults were obtained from the CDC PLACES data portal [21]. These estimates were based on results from BRFSS questions that ask whether individuals have ever been told by a physician or a health professional that they have diabetes or high blood pressure [22]. PLACES generates estimates at the census tract level using multilevel regression and poststratification that incorporates BRFSS results and the US Census Bureau’s American Community Survey data [23].

### Census Tract–Level Variables

Patient addresses were geocoded to a census tract at each of the MNEHRC health care systems. Census tracts for addresses that did not successfully geocode were assigned based on population ratios using a zip code to a census tract crosswalk from the US Department of Housing and Urban Development [24]. Census tract–level sociodemographic variables were obtained from multiple sources. Social Vulnerability Index (SVI), a measure “designed to identify and quantify communities experiencing social vulnerability,” was obtained from the CDC [25]. For analytic purposes, the continuous SVI was categorized based on quartiles, with SVI 1 referring to those in the lowest quartile of vulnerability. Rurality was assigned based on the US Department of Agriculture’s Economic Research Service rural-urban commuting area codes and the percentage of individuals living in rural areas from the US Census American Community Survey [19]. Census data were used to determine the age, race distribution, and the percentage of the population with a high school degree or General Educational Development [20].

### Analyses

Statewide population counts for various demographic subgroups were tabulated from MNEHRC’s 2020 HTAC data and compared with corresponding estimates from the 2020 census. County population counts from HTAC data were also compared with 2020 Census counts to assess coverage at the county level.

MNEHRC estimates of census tract hypertension and diabetes prevalence were calculated using 2021 and 2022 HTAC data, respectively. The obtained estimates were compared with CDC PLACES prevalence estimates, both of which were scaled 0 to 100. To assess agreement, the relative difference between the MNEHRC and PLACES estimates was computed. Bland-Altman plots were created to visualize agreement and systematic bias across all prevalence values.

Associations between tract demographic and socioeconomic characteristics and the prevalence of diabetes and hypertension were estimated separately for the 2 sources using linear regression. Predictors measured as proportions (eg, age >65 years, race, and poverty level) were modeled as a percentage of census tract population with a range from 0 to 100. Analyses were conducted using R (version 4.4.2; R Foundation for Statistical Computing).

### Ethical Considerations

HTAC is a public health surveillance project conducted in partnership with the Minnesota Department of Health and local public health agencies and, therefore, was not considered research per 45 CFR 46.102(l)(2). Therefore, it was not subject to institutional review board approval [26].

## Results

**Figure 1:**
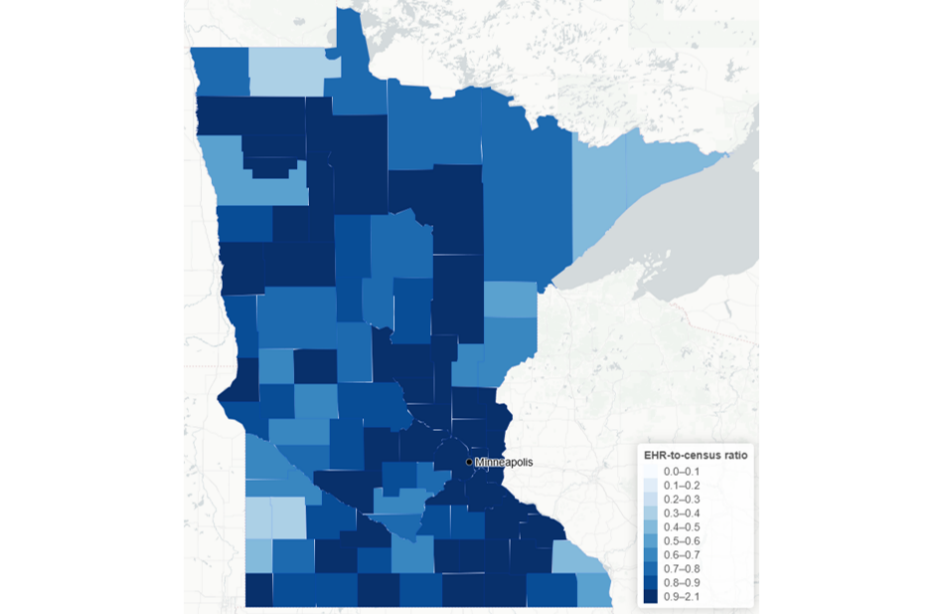
Ratio of 2020 Minnesota Electronic Health Record Consortium patient count to census population estimate, by county. EHR: electronic health record.

The 2020 census estimate for the population of Minnesota was 5,707,254. A total of 5,271,191 (92.4% of the census estimate) unique individuals visited 1 of the 11 MNEHRC health care systems in 3 years (2018-2020; Table 1). The ratio of MNEHRC patients to the Minnesota statewide census estimate was higher for female individuals (0.97) than for male individuals (0.88) and higher for the older age groups (65 years and older: 1.05) than for the younger age groups (0-17 years: 0.83). The MNEHRC patient-to-census ratio also differed by race—the ratio was highest for Black Minnesotans (1.17) and lowest for American Indian and Alaska Native Minnesotans (0.68). The MNEHRC data also had a greater number of individuals with missing, other, or unknown race (MNEHRC-to-census ratio 1.31). Coverage by county was generally consistent across the state, with a median MNEHRC patient-to-census estimate ratio of 0.89 (IQR 0.73-0.96; Figure 1).

**Table 1:** The 2020 Minnesota Electronic Health Record Consortium (MNEHRC) and census population estimates for Minnesota.

Characteristic		MNEHRC^a^, n; census^b^, n; ratio				
		All ages^c^	Aged 0-17 years^c^	Aged 18-44 years^c^	Aged 45-64 years^c^	Aged >65 years^c^
**Sex**						
	Male	2,482,165; 2,835,448; 0.88	557,640; 673,827; 0.83	857,548; 1,012,726; 0.85	612,529; 714,245; 0.86	454,448; 434,650; 1.05
	Female	2,789,026; 2,871,046; 0.97	531,812; 643,634; 0.83	1,027,682; 992,022; 1.04	688,909; 720,747; 0.96	540,623; 514,643; 1.05
**Race or ethnicity**						
	American Indian or Alaska Native	38,614; 57,046; 0.68	9944; 17,486; 0.57	16,504; 21,439; 0.77	8745; 12,859; 0.68	3421; 5262; 0.65
	Asian or Pacific Islander	259,865; 300,081; 0.87	74,740; 83,539; 0.89	113,407; 140,585; 0.81	50,488; 56,245; 0.90	21,230; 19,712; 1.08
	Black	460,302; 392,850; 1.17	148,679; 135,497; 1.10	200,991; 161,303; 1.25	83,392; 72,834; 1.14	27,240; 23,216; 1.17
	Hispanic	256,188; 345,640; 0.74	81,608; 126,873; 0.64	116,815; 150,957; 0.77	46,029; 54,280; 0.85	11,736; 13,530; 0.87
	White	3,923,405; 4,353,880; 0.90	690,332; 844,827; 0.82	1,311,314; 1,438,283; 0.91	1,048,115; 1,197,790; 0.88	873,644; 872,980; 1.00
	Other	336,761; 256,997; 1.31	85,216; 109,239; 0.78	128,012; 92,181; 1.39	65,072; 40,984; 1.59	58,461; 14,593; 4.01

^a^MNEHRC: Minnesota electronic health record consortium.

^b^Population estimates from the Minnesota statewide 2020 census.

^c^The values in all the columns indicate MNEHRC patients, Minnesota statewide 2020 census estimate, and the ratio of MNEHRC patients to the Minnesota statewide census estimate.

**Figure 2:**
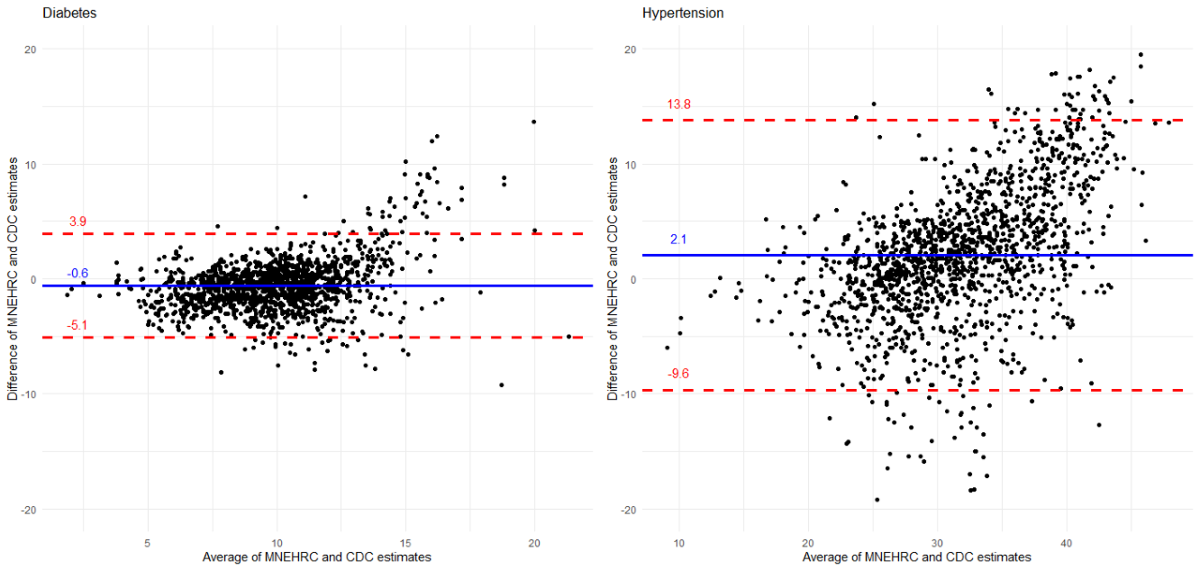
Bland-Altman plots comparing diabetes and hypertension prevalence estimates from the Centers for Disease Control and Prevention (CDC) Population Level Analysis and Community Estimates and cross-sectional electronic health record data from the Minnesota Electronic Health Record Consortium (MNEHRC) using census tract–level data.

**Figure 3:**
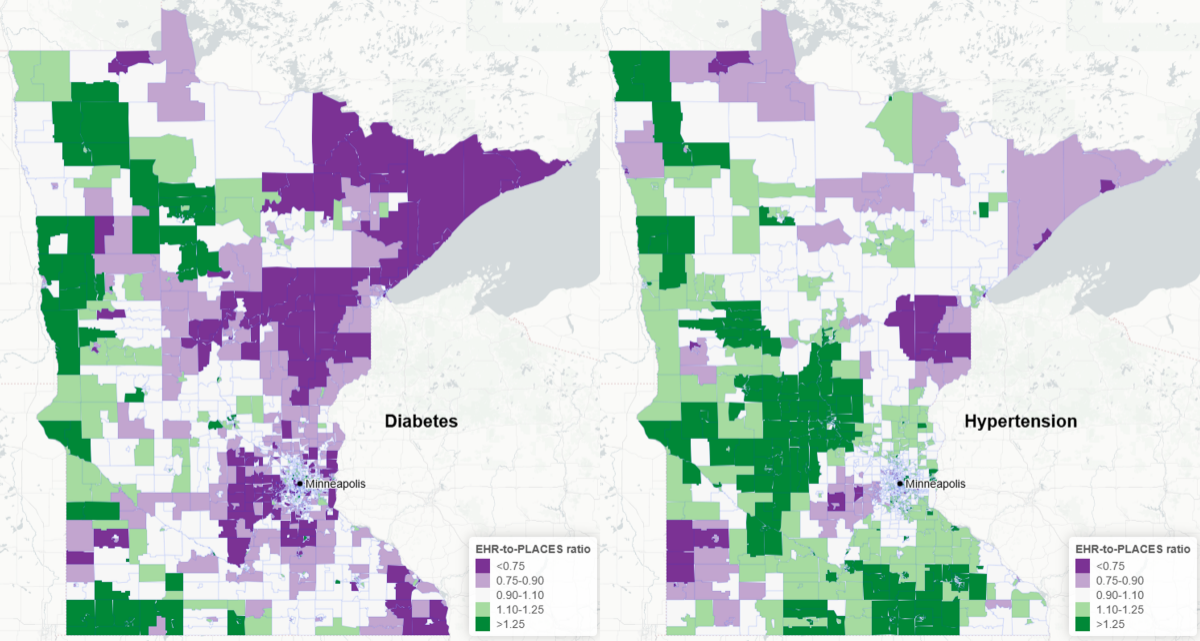
Ratio of Minnesota Electronic Health Record Consortium (MNEHRC) to Centers for Disease Control and Prevention Population Level Analysis and Community Estimates (PLACES) prevalence estimates for diabetes and hypertension at the census tract level. Blue represents census tracts where the MNEHRC estimate is lower than the PLACES estimate. Green represents census tracts where the MNEHRC estimate is higher than the PLACES estimate. White represents census tracts where the 2 estimates are within 10% of each other. EHR: electronic health record.

According to the MNEHRC data, the percentage of adults in Minnesota with diabetes in 2022 was 9.5% (415,914/4,376,805), and the percentage with hypertension in 2021 was 32.2% (1,365,413/4,243,000). Crude prevalence estimates from PLACES data for diabetes were 9.9% (435,481/4,389,028) and for hypertension were 29.9% (1,311,459/4,389,028). There was a moderate level of concordance between the prevalence of diabetes and hypertension at the census tract level among adults comparing MNEHRC and PLACES estimates (Figure 2). Relative to the PLACES estimates, MNEHRC estimates tended to be higher in high-prevalence census tracts. This was particularly true for hypertension. The percentage of census tracts where the MNEHRC estimate was within 10% of the PLACES estimate was 40.3% (605/1500) for diabetes and 42.3% (635/1500) for hypertension. The percentage of census tracts where the MNEHRC estimate was within 25% of the PLACES estimate was 77.9% (1168/1500) for diabetes and 79.7% (1195/1500) for hypertension (Figure 3).

Area-level factors associated with diabetes and hypertension prevalence were generally similar in linear regression models for MNEHRC and PLACES (Table 2). Factors associated with higher prevalence in all models included a higher proportion of persons aged 65 years and older, a higher SVI, nonurban census tracts, and a lower proportion of individuals with at least a high school degree or General Educational Development. A higher proportion of Black individuals in a census tract was associated with lower hypertension prevalence in MNEHRC models but higher diabetes and hypertension prevalence in PLACES models. SVI was a stronger predictor of diabetes and hypertension in MNEHRC models compared with PLACES models. Model *R*^2^ for diabetes was 0.46 in the MNEHRC model and 0.75 in the PLACES model; for hypertension, model *R*^2^ was 0.52 in the MNEHRC model and 0.81 in the PLACES model.

**Table 2:** Census tract factors associated with hypertension and diabetes prevalence estimates from the Centers for Disease Control and Prevention Population Level Analysis and Community Estimates (PLACES) and a cross-sectional study using electronic health record data from the Minnesota Electronic Health Record Consortium (MNEHRC).

			MNEHRC, coefficients (95% CI)^a^		MNEHRC, *P* value		PLACES, coefficients (95% CI)^b^		PLACES, *P* value
**Hypertension**									
	Individuals aged >65 years (%)	0.54 (0.48 to 0.60)		<.001		0.61 (0.59 to 0.64)		<.001	
	Black (%)	–0.10 (–0.14 to –0.06)		<.001		0.07 (0.05 to 0.09)		<.001	
	American Indian or Alaska Native (%)	–0.12 (–0.18 to –0.05)		<.001		0.05 (0.03 to 0.08)		<.001	
	Asian or HPI^c^ (%)	–0.08 (–0.13 to –0.03)		.001		–0.05 (–0.07 to –0.03)		<.001	
	SVI^d^ 2 (vs SVI 1)	1.62 (0.80 to 2.43)		<.001		0.07 (–0.28 to 0.41)		.70	
	SVI 3 (vs SVI 1)	2.19 (1.31 to 3.06)		<.001		0.55 (0.18 to 0.92)		.004	
	SVI 4 (vs SVI 1)	2.53 (1.43 to 3.63)		<.001		1.23 (0.76 to 1.70)		<.001	
	Exurban (vs urban)	4.16 (3.13 to 5.19)		<.001		3.00 (2.56 to 3.43)		<.001	
	Rural (vs urban)	4.25 (3.28 to 5.22)		<.001		3.31 (2.89 to 3.72)		<.001	
	Small town (vs urban)	5.98 (4.80 to 7.16)		<.001		2.03 (1.52 to 2.53)		<.001	
	Below poverty (%)	–0.05 (–0.08 to –0.03)		<.001		0.00 (–0.01 to 0.01)		.95	
	High school or General Educational Development (%)	–0.23 (–0.31 to –0.15)		<.001		–0.31 (–0.34 to –0.28)		<.001	
**Diabetes**									
	Individuals aged >65 years (%)	0.14 (0.12 to 0.16)		<.001		0.22 (0.21 to 0.24)		<.001	
	Black (%)	0.01 (–0.01 to 0.03)		.21		0.03 (0.02 to 0.04)		<.001	
	American Indian or Alaska Native (%)	0.07 (0.04 to 0.10)		<.001		0.07 (0.05 to 0.08)		<.001	
	Asian or HPI (%)	0.02 (–0.00 to 0.04)		.07		0.00 (–0.01 to 0.02)		.42	
	SVI 2 (vs SVI 1)	0.86 (0.52 to 1.20)		<.001		0.03 (–0.14 to 0.20)		.72	
	SVI 3 (vs SVI 1)	1.51 (1.14 to 1.87)		<.001		0.29 (0.11 to 0.48)		.002	
	SVI 4 (vs SVI 1)	1.88 (1.42 to 2.34)		<.001		0.46 (0.23 to 0.70)		<.001	
	Exurban (vs urban)	1.00 (0.57 to 1.43)		<.001		1.23 (1.01 to 1.45)		<.001	
	Rural (vs urban)	2.29 (1.89 to 2.70)		<.001		1.42 (1.22 to 1.63)		<.001	
	Small town (vs urban)	1.93 (1.44 to 2.43)		<.001		0.93 (0.68 to 1.18)		<.001	
	Below poverty (%)	–0.01 (–0.02 to –0.00)		.008		0.01 (0.00 to 0.01)		<.001	
	High school or General Educational Development (%)	–0.16 (–0.19 to –0.13)		<.001		–0.19 (–0.21 to –0.18)		<.001	

^a^Model *R*^2^ for hypertension = 0.52; Model *R*^2^ for diabetes = 0.46.

^b^Model *R*^2^ for hypertension = 0.81; Model *R*^2^ for diabetes = 0.75.

^c^HPI: Hawaiian or Pacific Islander.

^d^SVI: Social Vulnerability Index.

## Discussion

In this analysis comparing Minnesota statewide EHR data with census and CDC PLACES data, we demonstrated that population estimates from EHR data and the census are generally similar, though there are important differences by age, sex, and race. This finding has important implications for public health data; when possible, it is preferable to obtain numerators and denominators from the same source to avoid overestimating or underestimating prevalence or uptake of services. We also demonstrated moderate concordance between small-area prevalence estimates from EHR data and CDC PLACES. However, we did observe a greater than 25% relative difference in estimates for 22% of census tracts for diabetes and 20% of census tracts for hypertension. These results indicate the need for multiple data sources to accurately estimate small-area prevalence for chronic conditions.

Our results add to the literature evaluating the accuracy of BRFSS-based estimates. In the Boston validation study, BRFSS estimates were compared with estimates from a survey of 7340 individuals in 15 neighborhoods [27]. The BRFSS model estimates of diabetes prevalence were within the 95% CIs of the Boston survey for 80% of the neighborhoods. In the Missouri County-Level Study, 50,690 adults were surveyed across 115 counties [28]. The correlation between the BRFSS estimate for diabetes prevalence at the county level and the Missouri County-Level Study estimate was only 0.51. These prior studies were limited in that they compared estimates between 2 surveys. The results of this study allow a comparison between BRFSS and an estimate from a different data source, namely, EHR data.

Differences in population estimates between MNEHRC and the census may be due to multiple factors. Census undercounting may lead to a lower estimate of the number of Black Minnesotans. American Indian and Alaska Native Minnesotans may receive care at facilities that are not included in the MNEHRC, such as Indian Health Service clinics. The causes of differences in disease prevalence estimates between MNEHRC and PLACES are less apparent. PLACES may be inaccurate in certain census tracts due to their low survey rate, which necessitates modeling-based prevalence estimates. This may have led to some inaccuracies in prevalence estimates, which may be more apparent for the high-prevalence census tracts, as we observed with hypertension. Notably, the relationship between census tract characteristics and disease prevalence differed between MNEHRC and PLACES. A higher proportion of Black and American Indian or Alaska Native residents was associated with lower hypertension prevalence in MNEHRC data but higher hypertension prevalence in PLACES. This could represent differential access to care, lower rates of diagnoses, or PLACES model assumptions—such as age distribution—that are inaccurate in Minnesota. SVI was more strongly associated with hypertension and diabetes prevalence in MNEHRC models than in PLACES models, which may be due to a stronger association between SVI and prevalence in Minnesota compared with national estimates. The higher overall *R*^2^ in the PLACES model is expected as those estimates are inherently model based using census tract characteristics.

Estimating small-area prevalence using EHR data has several potential advantages. First, EHR data can capture nearly the entire population. In our MNEHRC data, more than 90% of the census-estimated population had an encounter at a participating health care system within a 3-year period. Comparatively, surveys collect data from a small number of individuals within each census tract, and most census tracts are not represented in the National Health and Nutrition Examination Survey at all. This study included the availability of statewide data from a large, multi–health care system distributed EHR network that is scalable in other states. The number of adults in the EHR data for most census tracts is >75% of the census estimate for that population, which makes the EHR-based estimates robust. This allowed for, what we believe is, the first comparison of CDC PLACES– and EHR-based small-area estimates of diabetes and hypertension prevalence. Second, established data collection methods reduce the need for expensive and time-consuming survey collection and field work. Third, EHR data can be produced in near real time. While our analysis cannot comment on which approach (EHR based vs survey) is more accurate, it appears that using EHR data to assess area-level trends would provide similar information to survey-based data and reduce the risk of errors related to discrepancies between numerator and denominator sources. Both EHR data and survey data are prone to some misclassification. While inaccuracies in EHR data may be a particular concern for people who have infrequent health care use, a common concern with survey data is recall bias. Factors that would favor use of EHR data include (1) high percentage of population included in EHR data sources, (2) ability to use objective data to identify conditions (eg, blood pressure for hypertension and hemoglobin A_1c_ values for diabetes), (3) conditions where EHR data have a high sensitivity, (4) conditions with low prevalence that would require surveying thousands of individuals, (5) conditions that patients may be unlikely to report on surveys either due to stigma or because they are unaware they have the disease, (6) when disease prevalence estimates for key subpopulations would be informative, and (7) when assessing local temporal trends in disease prevalence.

Our study has important limitations. First, the EHR patient count may overestimate the population due to incomplete deduplication, though this is likely to be small. Second, EHR data are unable to account for out-migration, which might represent 1% to 2% of the effect on the population in any given year [29]. By contrast, the EHR patient count may underestimate the population due to individuals being seen by health care providers not included in MNEHRC and individuals who have not been seen by a health care provider in the last 3 years. Third, patients who never seek care may vary in important ways from health care–seeking patients, resulting in EHR-based population and prevalence estimates that do not reflect the entire population. By contrast, the census and PLACES data represent individuals who respond to surveys. Relatedly, both EHR estimates and survey estimates likely underestimate disease prevalence due to undiagnosed disease and lack of disease awareness, but EHR estimates may underestimate disease prevalence less than self-report. Fourth, our study does not allow us to identify a threshold of EHR data coverage above which it becomes more appropriate to use EHR data rather than survey data for public health decision-making. The results of this study are relevant to distributed data networks with robust geographic coverage, particularly those that use OMOP. Finally, our models were estimated via ordinary least squares regression, which assumes independence across census tracts. Because tracts are spatially correlated, uncertainty surrounding coefficient estimates is likely understated, although the estimates themselves are unbiased.

In conclusion, we identified important differences between EHR and census data by age, sex, and race. These differences should be considered in public health reporting when 2 different data sources are used for the numerator and denominator, as was frequently done during the COVID-19 pandemic, where vaccination rates were estimated using census denominators. The prevalence of diabetes and hypertension at the census tract level was generally concordant between MNEHRC and PLACES, but differences suggest that EHR data may help inform targeting of public health interventions to reduce prevalence and adverse outcomes from chronic conditions. Our analysis suggests that there are both similarities and important differences between small-area estimates derived from EHR and survey data. Such differences suggest that further research with validation against an assessment of all individuals using clinical gold standards is needed to determine the optimal collection method for local estimates of health conditions.

## Data Availability

The datasets generated or analyzed during this study are not publicly available as they include patient data from multiple health systems. Only aggregate results were shared centrally.

## Abbreviations

BRFSS: Behavioral Risk Factor Surveillance System
CDC: Centers for Disease Control and Prevention
EHR: electronic health record
HTAC: Health Trends Across Communities
MNEHRC: Minnesota Electronic Health Record Consortium
OMOP: Observational Medical Outcomes Partnership
PLACES: Population Level Analysis and Community Estimates
SVI: Social Vulnerability Index
